# Use self-gravitation traction to treat lumbar disc herniation: Study protocol for a double-center, single-blind randomized controlled trial

**DOI:** 10.1097/MD.0000000000031717

**Published:** 2022-11-25

**Authors:** Xi-Yun Zhao, Zhi-Peng Wang, Zhen Quan, Guo-Dong Gao, Hong-Wei Zhang, Xiao-Gang Zhang, Lin-Zhong Cao, Shuo Liu, Jin-Feng Li

**Affiliations:** a Clinical College of Chinese Medicine, Gansu University of Traditional Chinese Medicine, Lanzhou, China; b Spine Surgery, Affiliated Hospital of Gansu University of Traditional Chinese Medicine, Lanzhou, China.

**Keywords:** low back pain, lumbar disc herniation, protocol, randomized controlled trial, self-gravitation traction

## Abstract

Self-gravitation traction is 1 of the most popular treatments for lumbar disc herniation (LDH). This study aims to evaluate the effectiveness and safety of the self-gravitation traction device in the treatment of LDH and to confirm its positive treatment effect.

**Methodology:**

This trial is designed as a pragmatic double-center, single-blind, and 3-arm (1:1:1 ratio) randomized controlled trial. The recruited patients with LDH will be randomly allocated to the intervention (traction weight is 40% or 60% of its body weight) or control (traction weight is 20% of its body weight) group. Traction will be completed within 6 consecutive weeks (3 times a week), with 10 minutes of traction for the first 3 weeks, 20 minutes of traction for the next 3 weeks. After the experiment is completed, we will establish an experiment-related database. The software of SPSS, version 21 (SPSS Inc. Chicago, IL) will be used for statistical analysis, and measurement data will be expressed via mean and standard deviation (mean ± SD).

**Discussion:**

Once the trial is completed, we will publish the study in journals in both Chinese and English to promote the dissemination and use of the results. In addition, we also plan to promote the research results at various academic conferences both domestically and internationally.

## 1. Introduction

Low back pain (LBP) is 1 of the most common presenting complaints in the world, and about 70% to 80% of the population sustains an episode once in their lifetime.^[1,2]^ Upright walking human beings need to stand and sit for a long time, thus their lumbar vertebrae bear greater load that might lead to intervertebral disc degeneration and disorder of relevant motion segment structures, such as small joints, ligaments, and muscles. Consequently, the lumbar spine loses its mechanics balance and eventually leading to LBP.^[3,4]^

However, LBP is a clinical symptom rather than a specific diagnosis. LBP is the most common clinical manifestation of lumbar disc herniation (LDH). LDH represents a frequent disorder encountered in current clinical practice and is associated with significant disability and morbidity.^[5]^

Currently, non-operative treatment is the first-line treatment for most LDH patients.^[6,7]^ Complementary and alternative treatments are the main focus,^[8]^ such as physical therapy/exercises,^[9]^ spinal manipulation,^[10]^ traction (manual or mechanical),^[11]^ transcutaneous electrical stimulation,^[12]^ acupuncture,^[13]^ herbal supplementation, et cetera.^[14]^ Traction is the most popular treatment for LDH among all listed. Traction is a broad term that includes mechanical traction, automatic traction, manual traction, gravity traction, and water traction, et cetera.^[15–18]^ And, these traction treatment method varies in their traction force, traction frequency, treatment time, and duration of treatment. Apart from this, the therapeutic effectiveness of traction is not been recognized by everyone. For example, some studies believe that traction is not an effective treatment method for all LDH patients.^[19,20]^ Therefore, it is very necessary to develop a large-scale and more standardized randomized controlled trial (RCT) to evaluate the therapeutic effectiveness of traction on treating LDH patients.

Continuous and controllable lumbar traction can: reduce the pressure in the intervertebral disc; alleviate the pressure of the lumbar facet joints to correct the disorder of the facet joints, and balance the various muscle groups of the lumbar spine to achieve its effective biomechanics effects. Self-gravitation traction is 1 of them.^[21]^ Thus, our team has developed a self-gravitation traction device to achieve continuous and controllable lumbar traction. The aim of this RCT is to evaluate the effectiveness and safety of the self-gravitation traction device on treating LDH and to confirm the positive effect of the device on the treatment of LDH.

## 2. Methodology

The study protocol is approved by the Medical Ethical Committee of the Affiliated Hospital of Gansu University of Chinese Medicine in March 2020 (Number: [2020]08). The trial is prospectively registered with the Chinese clinical trial registry (ChiCTR2100045118).

### 2.1. Trial design

**Figure 1.**
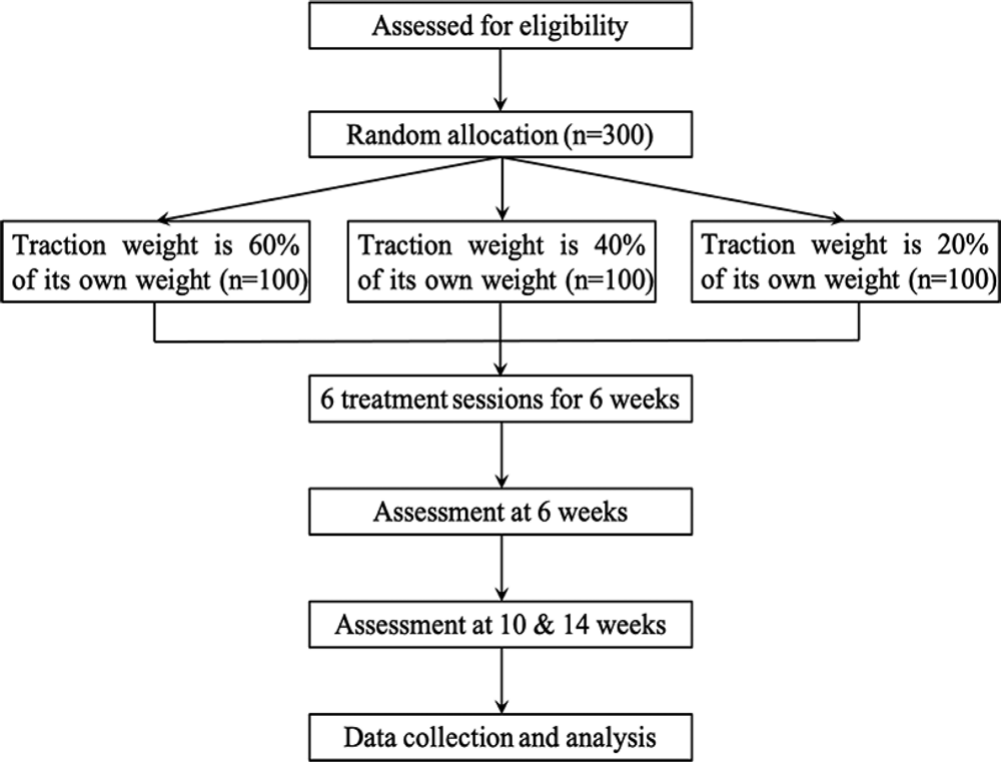
Study flowchart.

This trial is designed as a pragmatic double-center, single-blind, and 3-arm (1:1:1 ratio) RCT to evaluate the effects of self-gravitation traction on LDH patients. The trial is being conducted in 2 hospitals in Lanzhou, China (Affiliated Hospital of Gansu University of Chinese Medicine and Gansu Provincial Hospital of Traditional Chinese Medicine). The recruited LDH patients are randomly allocated into the intervention group (traction load is 40% or 60% of their body weight) or control group (Traction weight is 20% of its body weight).^[18,20,22]^ Traction treatment procedure is completed within 6 consecutive weeks (3 times a week), 10 minutes’ traction for the first 3 weeks, then increase to 20 minutes for the next 3 weeks. See details in Figure 1.

### 2.2. Participants

#### 2.2.1. *Eligibility*.

Adult men and women (aged 18‐50 years) who have been diagnosed with LDH using Lumbar MRI are recruited to participate in the study. These participants also need to meet the following 3 conditions: MRI result shows unilateral segmental nerve root compression; clinical LBP symptoms and sciatica have lasted for more than 3 months; volunteer to participate in the study and informed consent form is agreed and signed. All participants with any of the following conditions will be excluded from the study: Tumor, infection, or fracture-related LBP; has received surgical operations on the lumbar region; has neurological defects diseases; suffers from severe osteoporosis; suffers from mental illness, such as insomnia, depression, or anxiety; pregnant women or women whose maternity is within half a year; with severe cardiovascular disease or lung disease; with liver or kidney dysfunction; patients whose lumbar MRI/CT examination indicate lumbar disc prolapse (prolapse or upturn) or calcification.

#### 2.2.2. *Sample size*.

Prior to launch the study officially, a pilot feasibility study (unpublished material) is conducted. Sample size calculation is based on the effective size of the pilot feasibility study. Finally, the study plan is to recruit 300 patients and distribute them at a ratio of 1:1:1, with 100 cases in each group.

#### 2.2.3. *Recruitment and screening process*.

Orthopedist selects eligible LDH patients according to the inclusion criteria and exclusion criteria. Both outpatients and inpatients are recruited. Patients who meet the inclusion criteria are asked whether they are interested in participating in the study via face-to-face conversation or phone inquiry. Patients who agree to participate voluntarily are invited again to come to the hospitals for eligibility review. At the same time, patients are free to ask any additional questions about the study. At this point, patients who confirm to participate in sign a written informed consent, and signed participants receive an interview and complete a set of questionnaires.

### 2.3. *Randomization and blinding*

The study adopts a central stratification and block randomization design. Patient is randomized to the study once they meet the eligibility criteria in which randomization sequence will be generated through SPSS software, version 21 (SPSS Inc. Chicago, IL). The study site of each hospital receives its randomization lists once a week from the primary study center. The randomization sequence cannot be edited and is concealed from the researcher. The aforesaid process of randomization ensures the objectivity of the researcher and eliminates bias in allocating participants groups. After the completion of blind editing, a document is prepared to record the entire process of blind editing, and the 2 copies of the blind secret will be stored in the primary study center and the application hospitals. When the study is completed, the staff who saves the blind bottom from the primary study center will do the first unblinding to get the group code, such as the experimental group or the control group. Afterward, statical data analysis will be done accordingly. Once analysis is completed, the researcher of the application hospital conducts a second unblinding and learns the 3 groups under specific interventions.

### 2.4. Interventions

#### 2.4.1. *Monitor traction*.

**Figure 2.**
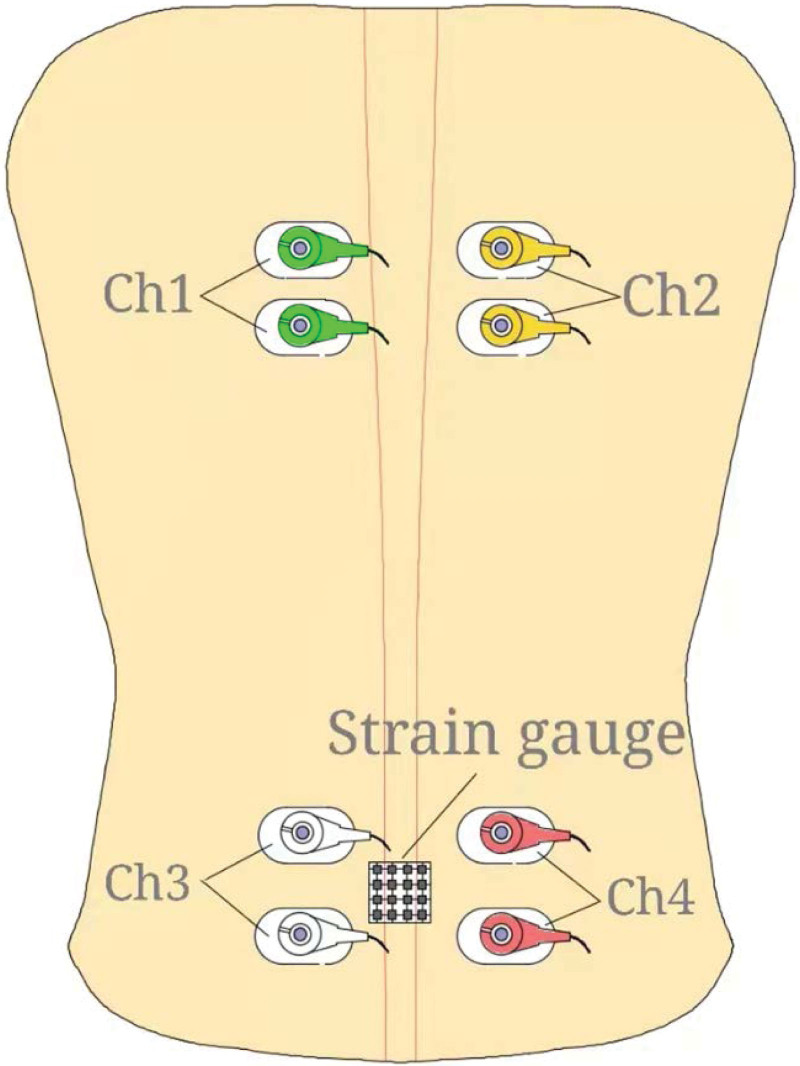
Electrode connection diagram.

Therapist will place 4 pairs of surface electrodes on the corresponding positions of the participant’s back muscles (muscle movement points) and place the electrodes parallel to the direction of the fiber (Fig. 2). Two electrodes will be separated by 2~3cm and connected to the 8-channel muscle. The surface electrical signal will be collected on the electrograph and connected to the computer analysis software to quantify the co-contraction performance of the muscle. At the same time, the electrocardiograph will be used to dynamically measure the heart rate to determine the patient’s relaxed state. Then, the therapist will use an adhesive to stick the strain gauge on the surface of the participant’s lumbar spine and connect it to a static resistance strain gauge and record the stress changes through a strain sensor (Fig. 2).

#### 2.4.2. *Phystrac mechanical traction therapy*.

**Figure 3.**
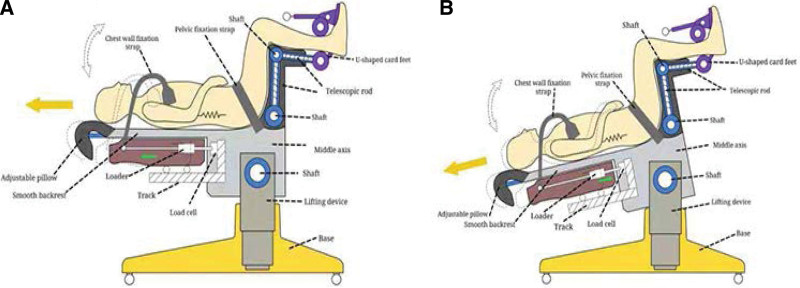
Schematic diagram of quantify traction and set traction angle.

Participants are in a supine position on the traction machine through adjustment, with hip and knee joints flexed at 90°, and double-fixed with a pelvic belt and ankle joint U-shaped feet (Fig. 3A). Start the weighing sensor system, apply mechanical load in advance through the axial pulling of the armpit on the horizontal plane, and will use the load cell to measure the mechanical traction. It is initially set as the preload, and on this basis, the shaft is gradually rotated to the head-down and the foot-high position, and the bodyweight will be used to complete the equivalent transformation of force (Fig. 3B). When the traction device rotates to a certain angle (the angle measuring instrument records the traction angle), the preload of the horizontal mechanical load will completely transform into its gravity. Controlled and quantified traction, continuous and repeated self-weight traction at this tilt angle.

### 2.5. *Observation indicators and outcomes*

#### 2.5.1. *Physical data*.

In the process of traction therapy, the following data needs to be recorded: use the load cell to record the traction load; use an infrared range finder to measure the moving distance of the body under traction; record the stretching distance of the lumbar spine on the skin surface through a strain sensor; record dynamic heart rate and surface electrical signal through electrocardiograph and 8-channel electromyography; use angle measuring instrument to record traction angle. All output data will be sorted and managed by a dedicated person.

#### 2.5.2. *Clinical outcomes*.

The outcome of treatment will be observed at the 4th week and 8th week after the whole course of treatment. Adverse events will be monitored throughout the study period. In addition, the following tests will be performed respectively at weeks 1, 6, 10, and 14: complete blood count; liver and renal function tests; blood coagulation test; urinalysis; and pregnancy test (if necessary).

The primary outcome will be a change in the intensity of low back pain. We plan to use the 100-mm visual analogue scale (VAS) to evaluate the intensity of low back pain: 0 means absence of pain, 100 means worst pain imaginable.^[23]^ We will ask participants to record their pain intensity within the past week by the 100-mm VAS at the following time points: pretrial screening, weeks 1 (baseline), week 6 (primary endpoint), and at weeks 10 and 14 (follow-up sessions). Change from baseline after 6 sessions of treatment will be compared between the groups as the primary outcome.

The secondary outcomes will be the Roland-Morris disability questionnaire (RMDQ), Oswestry disability index, 36-Item short form (SF-36) Health Survey, and adverse events.^[24–26]^ The RMDQ contains 24 sentences that people have used to describe themselves when they have low back pain, and participants check the sentences to describe their life on the day. The ODI questionnaire consists of 10 sections: pain; personal care; lifting; walking; sitting; standing; sleeping; sex life; social life; and traveling. We plan to use the RMDQ to assess the dysfunction in daily life and use the ODI to assess the inability to function in daily life due to low back pain at weeks 1, 6, 10, and 14. The SF-36 is a very popular instrument for evaluating Health-Related Quality of Life. The SF-36 measures 8 scales: physical functioning, role physical, bodily pain, general health, vitality, social functioning, role emotional, and mental health. We will ask participants to record their quality of life by the 100-mm VAS at the following time points: weeks 1, 6, 10, and 14.

### 2.6. *Statistical analysis*

After the experiment is completed, we will establish an experiment-related database. The software of SPSS, version 21 (SPSS Inc. Chicago, IL) will be used for statistical analysis, and measurement data will be expressed by Mean and Standard Deviation (mean ± SD). The independent t-test and Chi-square test will be used to compare differences in general characteristics between the groups. All statistical analyses will be performed using PASW statistics 18 for Windows, and the statistical significance level will be considered to be 0.05 (2-sided), with 95% confidence intervals.

## 3. Discussion

As a high incidence disease, intervertebral disc herniation is a spinal condition that can cause back pain and/or radiculopathy.^[27]^ Traction is an effective treatment, but it is not standardized at present. Therefore, it is very important to formulate the optimal traction dose and traction parameters. The self-gravitation traction device independently developed by our team completely converts the preload loaded by horizontal machinery into its gravity control and quantified traction force through the equal conversion of force, to complete the accurate control and quantification of traction force. It can carry out continuous traction for a certain time to make up for the defects of individual differences and poor tissue compliance during continuous mechanical traction, to provide a reliable and accurate research method for lumbar traction in the treatment of LDH.

After the completion of this RCT, we will publish the study in journals in both Chinese and English to promote the dissemination and use of the results. In addition, we also plan to promote the research results at various academic conferences both domestically and internationally.

## Acknowledgements

We thank LetPub (www.letpub.com) for its linguistic assistance during the preparation of this manuscript.

## Author contributions

**Conceptualization:** Zhi-Peng Wang.

**Data curation:** Guo-Dong Gao, Jin-Feng Li, Zhen Quan.

**Formal analysis:** Lin-Zhong Cao.

**Investigation:** Jin-Feng Li.

**Methodology:** Shuo Liu.

**Resources:** Xiao-Gang Zhang.

**Software:** Xiao-Gang Zhang.

**Supervision:** Hong-Wei Zhang.

**Writing – original draft:** Xi-Yun Zhao.

**Writing – review & editing:** Xi-Yun Zhao, Zhi-Peng Wang.
